# Patients profiling for Botox® (onabotulinum toxin A) treatment for migraine: a look at white matter lesions in the MRI as a potential marker

**DOI:** 10.1186/2193-1801-2-377

**Published:** 2013-08-10

**Authors:** Anja Bumb, Burkhard Seifert, Stephan Wetzel, Reto Agosti

**Affiliations:** Headache Center Zürich Hirslanden, Forchstrasse 424, 8702 Zollikon, Switzerland; Swiss Neuro Institute Hirslanden Zürich, Zürich, Switzerland; Department of Biostatistics, University of Zürich, Zürich, Switzerland; Department of Neuroradiology Hirslanden Zürich, Zürich, Switzerland; University of Basel, Zürich, Switzerland

**Keywords:** Botox®, WML, MRI

## Abstract

**Background:**

To evaluate if white matter lesions (WML) on MRI can be a potential marker for onabotulinum toxin A (Botox®) treatment success in migraine, given the limited response rate and high costs per treatment.

**Methods:**

Retrospective data base and MRI analysis of 529 migraineurs who received Botox® between 2002 and 2009. Responders were defined as patients who underwent three or more treatments, whereas non-responders had only one or two treatments. MRIs were analysed on axial T2 and coronar FLAIR (fluid attenuated inversion recovery) sequences for the presence of WML. Statistical analysis was done with the Chi-Square-Test and the Mann–Whitney-U-Test.

**Results:**

Of 529 Botox® treated migraineurs, 111 patients had a MRI. Of these 111 patients, 47 were responders, 64 non-responders to Botox®. Response rate to Botox® in migraineurs with WML was 55.3%, in migraineurs without WML 44.7%. In the investigated items “age”, “age at onset”, “gender”, “attack duration”, “frequency”, “aura”, “WML”, “size of WML”, we found no statistical significant difference between the two groups. 55% of the responders and 50% of the non-responders showed WML. All WML were located supratentorially, anteriorly, mostly of small size (3–5 mm).

**Conclusion:**

WML on MRIs cannot serve as a marker to predict a positive response to Botox®.

## Background

Migraine is a primary headache disorder. According to the WHO, the lifetime prevalence of migraine in Europe and North America is 6% in men and 15-18% in women for one year (Natoli et al. 2010; Leonardi & Mathers 2000). Several large longitudinal studies regarding migraine prevalence exist, the AMPP (American Migraine Prevalence and Prevention) and the Norwegian HUNT study (Munakata et al. 2009; Linde et al. 2010), indicating a slight increase in migraine over the last years. Improved prevention treatment is needed, with higher efficacy, causing fewer, at best no side-effects.

An approach for this kind of prevention, might be the use of onabotulinum toxin A (Blumenfeld 2003). Botulinum toxin is used since the early 70s for medical purposes, first to correct strabism and later to treat focal dystonias, spasticity, hyperhidrosis and many other disorders (Lukban et al. 2009; Rosales & Chua-Yap 2008; Binder et al. 2000). Since 2010, based on the two PREEMPT-studies (Phase III Research Evaluating Migraine Prophylaxis Therapy), onabotulinum toxin A is registered for the indication chronic migraine in the USA and since 2011 in Great Britain and the European Community.

Botox® (Allergan, Inc., Irvine, CA) mediates its postulated mechanism of action in migraine by inhibiting the release of nociceptive agents, such as glutamate, substance P, calcitonin gene-related peptide and acetylcholine (Durham & Cady 2004; Gupta et al. [Bibr CR18][Bibr CR19]). The advantage of a treatment with Botox® is the good tolerability, the lack of side-effects and the therapeutic effect over three to six months. The success rate varies between 30% and 50% (Dodick et al. 2010). The main disadvantage are the high costs of one Botox® treatment that are mostly not reimbursed. However, patients with chronic migraine, suffering predominantly from unilateral headache, presence of scalp allodynia and pericranial muscle tenderness, seemed to show a rather good response (Blumenfeld et al. 2010a; Robertson & Garza 2012).

It is known that subjects with migraine are at higher risk of having WML on the MRI than those without migraine (Mathew et al. 2008). Several studies, such as the CAMERA (Cerebral Abnormalities in Migraine, an Epidemiologic Risk Analysis) study showed that migraineurs, notably those with aura, had a higher prevalence of subclinical infarcts in the posterior circulation territory. Higher risk of lesions was present in those with higher attack frequencies or longer migraine history (Richard et al. 2004). The etiology of the WML remains unclear. A possible pathological mechanism is ischemia, maybe mediated through cortical spreading depression that causes disruption of the blood brain barrier (BBB) through a matrix metalloproteinase-9-dependent cascade mechanism, which may result in local tissue damage (Woods et al. 1994; Ayata et al. 2006).

The aim of our study was to investigate, if WML on MRI scans can serve as a marker to evaluate in advance the success of a treatment with Botox® in migraineurs. We focused on the two groups “responders” and “non-responders” to Botox® and tried to find some predicting differences in these two groups regarding success rate to Botox®.

The association between Botox® and WML in the MRI has until now not yet been studied.

## Methods

Our center is specialised in the diagnosis and treatment of headache disorders with 2.000 new headache patients per year and is experienced in the use of Botox® for migraine since 2002.

### Clinical parameters

In a retrospective fashion, 529 patients were identified from our database between 1 January 2002 and 1 July 2009, having received Botox® treatment for migraine. Of these 529 patients, 111 had a MRI scan. Data were collected of these 111 patients. The database contained name, gender, date of birth , migraine history, chronification, number of Botox® treatments, date of MRI scan, number, localization and size of WML.

Responders to Botox® were defined by us as patients who underwent three or more treatments, non-responders one or two treatments.

The Botox® therapy followed the recommendations of the PREEMPT trials (Neema et al. 2009). However, we used a smaller dose of Botox® (100 IU versus 155 IU), mainly because of the costs that are predominantly paid by the patients themselves. And the application sites with the dose of Botox® for each muscle were slightly divergent from the PREEMPT paradigm. They are shown in Table 1.

**Table 1:** **Botox® scheme for migraine at our center**

	Right injections	IU	Left injections	IU	Total IU
Muscle					
Frontal	2	2.5	2	2.5	10
Corrugator	1	2.5	1	2.5	5
Procerus	1	2.5	1	2.5	5
Temporal	3	5	3	5	30
Suboccipital	1	2.5	1	2.5	5
Semispinal	1	2.5	1	2.5	5
Splenius	1	2.5	1	2.5	5
Trapezius	6	2.5	6	2.5	30
Occipital	1	2.5	1	2.5	5
Total	17	25	17	25	100

### Imaging

MRIs were available electronically from the hospital radiology system. All scans had been performed on 1.5 Tesla or 3 Tesla MR tomographs, according to a standardized migraine protocol. 58 MRIs with reported WML and 53 MRIs with no reported WML were analysed, on coronar FLAIR sequences and on axial T2 sequences, in maximal 5 mm slices. WML were classified as small (3–5 mm), medium (6–9 mm) or large (>10 mm). Total number of lesions was recorded. The distribution was classified in supratentorial or infratentorial. If supratentorial, in anterior or posterior, with cut at the middle of corpus callosum.

Each WML analysis was performed independently by two neurologists, each blinded to the history of the patient. In case of disagreement between the two readers, a consensus was achieved by discussion.

Statistical analysis followed the statistical program SPSS (Superior Performing Software System). The Chi-Square-Test was applied for the items gender, duration, frequency (episodic vs. chronic), aura and non-parametric values such as age, age at onset, WML, were analyzed by the Mann–Whitney-U-Test.

## Results and discussion

### Clinical parameters

In the current group of 111 Botox® treated patients, 47 have been responders and 64 non-responders. We found in none of the investigated parameters a statistical significance to characterize or distinct responders from non-responders, details are shown in Table 2. Both groups have been in the middle ages, with disease onset as young adults. Women were predominant in both groups. The presence of aura was not predictive to a Botox® response, neither the type of migraine “episodic” or “chronic”.

**Table 2:** **Responders versus non-responders**

	Responders	Non-responders	p-value
	to Botox®	to Botox®	
Age (mean)	47	52	0.07 ns
Age at onset (mean)	21	21	0.912 ns
Gender (m/f) %	15/85	23/77	0.265 ns
Lifetime migraine (years)	26	31	0.255 ns
Chronic migraine %	66	62.5	0.708 ns
Aura %	60	52	0.402 ns
WML %	55	50	0.579 ns
WML small (mean per person)	2.3	2.9	0.897 ns
WML medium (mean per person)	0.2	0.2	0.875 ns
WML large (mean per person)	0.02	0.03	0.750 ns

### Imaging

**Figure 1:**
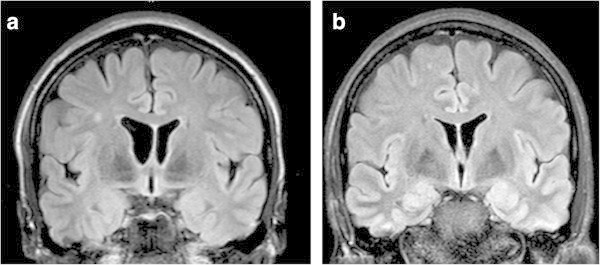
**Coronar brain MRI slices (FLAIR), in (a), on the left side, with one WML in a responder and in (b), on the right side, with three WML in a non-responder.**

The analyzed WML in the MRIs followed no pattern to permit a conclusion for a positive response to a Botox® treatment. WML were absent in 45% of the responders, present in 55%. In the non-responders, WML were absent in 50%, present in 50%. Mean lesion load of small-size- WML in responders was 2.3 per person, in non-responders 2.9 per person. Mean lesion load of medium-size- WML was 0.2 in both groups per person and of large-size- WML was 0.02 per person in responders and 0.03 per person in non-responders. Figure 1 shows the typical distribution of WML in our Botox®-migraine-population on MRI, in responders and non-responders. They are located supratentorially and anteriorly, mostly of small size.

The aim of our study was to find a marker of response to Botox®, in order to optimize treatment of migraine patients in clinical practice.

The response rate to Botox® in the treatment of migraine is generally in a range of 30% to 50% (Blumenfeld et al. 2010a). In our study, the response rate to Botox® in migraineurs with WML was 55.3%, in migraineurs without WML 44.7%. Our definition for a response to Botox® was pragmatically by assigning migraine patients to the number of Botox® treatments, so that responders were defined as migraineurs with three or more treatments and non-responders as migraineurs with one or two treatments. This endpoint has not been used before and is a simplified response criterion that is easily generated even in a retrospective analysis. The more sophisticated endpoints, usually generated in migraine prophylaxis studies, such as PREEMPT, are typically not obtainable in clinical practice. Nevertheless, our response rates are in the range of those in standard clinical trials, such as the pooled analysis in the two PREEMPT studies, with a 50% response rate of Botox® against placebo. This response rate was measured by reduction in mean frequency of headache days, headache episodes and improvement of patients’ functioning, vitality, psychological distress and overall, quality of life (Blumenfeld et al. 2010a). In our study, the gain of quality of life was assessed in the regular clinical follow-ups of the patients and documented in the patients’ history, but not by specific questionnaires or daily phone calls to a trial center.

Since WML are associated with the so called burden of disease in migraine sufferers, we attempted to analyze our migraine Botox® population with respect to WML as a possible predictor. The clinical importance of WML on MRI scans in different medical conditions has been shown before. WML serve as a biomarker for an increased risk of cerebrovascular events and predict a higher risk of stroke, dementia and death (Bigal 2010). However, in our study, the comparison of the two groups Botox®-responders and Botox®-non-responders showed no difference in the investigated items. So, our initial hypothesis, that white matter lesions could serve as a biomarker to predict a better response to Botox® in migraine treatment was disproved. The appearance of WML is not related to success or failure to a Botox® treatment, nor can presence or absence of WML predict the outcome of a treatment with Botox®.

In general, the meaning of these WML in migraineurs is unclear (Colombo et al. 2011) and the clinical importance often remains meaningless. However, before focussing on details in the discussion of WML, some basics have to be taken into account. Steady improvements of MRI techniques, with increasing use of 3T MRI, even 7T in some centers, instead of 1.5T MRI, show differences in the outcome of WML. So, for example in the study of Neema et al., realized in healthy volunteers (Neema et al. 2009), WML were seen three times more on FLAIR sequences of 3T MRIs than on FLAIR sequences of 1.5T MRIs. Sometimes, Virchow-Robin (VR) spaces may contribute to some confusion in analyzing WML. They have to be well distinguished from WML. VR spaces surround the walls of vessels and course from the subarachnoid space to the brain parenchyma. With advancing age, they become more frequent and larger in size (>2 mm). The signal intensity of VR spaces is identical to that of cerebrospinal fluid on all MR sequences. So, the FLAIR sequence is ideal, to distinct VR spaces from WML in difficult situations (Kwee & Kwee 2007).

In migraine, WML are more often seen in chronification (Schwedt & Dodick 2009; Debette & Markus 2010). So, chronic migraineurs with a longer duration of migraine and a higher attack frequency might contribute to a higher amount of WML (Schmitz et al. 2008). This is confirmed in our study, where WML appear to a higher amount in chronic migraine and less in episodic migraineurs. These findings are consistent with the concept of migraine chronification that can be seen on different levels, first in clinical transformation (increased frequency), physiologic transformation (allodynia, central sensitization) and, finally, anatomic progression with presence of WML (Aguggia & Saracco 2010; Bigal & Lipton 2008).

The distribution of WML in migraine has already been a subject of interest in various studies. Especially in migraine with aura patients, lesions in the deep white matter of the brain were detected, mainly in the frontal lobes. The type of aura symptoms did not correlate with the location of WML in the brain (Rossato et al. 2010). However, in some studies like the CAMERA-study, subclinical brain infarcts were located exclusively in the posterior circulation territory, especially in the cerebellum. The authors assumed an ischaemic origin through hypoperfusion and/or embolisms. Right-left-shunts of persistent foramen ovale as potential origin were not investigated. The lesions had a diameter of up to 7 mm. These lesions were mostly seen in female migraine with aura patients (8%) with higher attack frequency (Kruit et al. 2009). In the study of Scher et al., investigating the association of migraine headache and brain infarcts, an increased risk of cerebellar infarcts in middle aged women with migraine with aura was found (Scher et al. 2009). In our study, all WML were located supratentorially and anteriorly, mostly of small size. However, we did not find any difference in responders or non-responders concerning age, gender or aura.

The etiology of WML remains unclear. An ischemic origin has been postulated in most publications (Bigal 2010). It could be conceivable, that damage to the white matter may also happen by excitatory neurotransmitters, especially glutamate and ATP, which can result also in ischemic lesions. A disruption of glutamate homeostasis can be deleterious to neurons and oligodendroglia (Matute 2011). Furthermore a glutamate induced activation of phospholipase A2, has been attributed to play a major role in the neurotoxicity encountered during brain ischemia (Khanna et al. 2010).

In summary, different pathological mechanisms can be responsible for the presence of WML. First, an inflammatory origin, seen in autoimmune disorders (for example, multiple sclerosis, vasculitis (Chen et al. 2010), lupus erythematodes) or in infectious diseases like borreliosis. Second, an ischemic origin, like in cerebro-vascular diseases (Bonati et al. 2005) such as brain infarcts or inherited metabolic disorders like Fabry disease. Third, even “older age” without presenting any cerebrovascular risk factors is enough for developping WML, as shown in a study by Chowdhury et al. (Chowdhury 2011), including patients with a mean age of 61.7 years. Fourth, vascular dementias, Alzheimer’s disease and cerebral amyloid angiopathy can contribute to WML. Deposition of amyloid in the arteries, resulting in hypoperfusion can result in WML. In these conditions, the leading clinical symptoms of the WML are cognitive decline and symptomatic depressive states. Fifth, in mood disorders, especially bipolar disorders, WML are often present. They have been associated with the emotional and cognitive symptoms in bipolar disorder, caused by disruption of the fibers from the amygdala to other brain regions, leading to the presence of WML. It has even been discussed that WML could serve as a biomarker for the disturbances in mood and cognition in bipolar disorder (Benedetti et al. 2011; Gunde et al. 2011). Sixth, an cardioembolic mechanism of WML, caused by a right-to-left-shunt from a persistent foramen ovale, atrial fibrillation, can be a possible etiologic mechanism (Park 2011).

But not only the origin of the WML is heterogeneous, but as well their evolution. So, a progression of WML in healthy elderly people (mean age 71 years) was demonstrated in a study over three years (Sachdev et al. 2007). In contrast, a case report of a chronic migraine patient, showed a disappearance of WML in control MRIs over 5 months (Rozen 2010).

The precise mechanism of Botox® as headache prophylaxis is not fully elucidated, human and animal studies have shown that Botox® blocks release of neurotransmitters associated with the genesis of pain. The heavy chain of botox A binds to a ganglioside receptor in the plasma membrane of the presynaptic nerve terminal. This leads to receptor mediated endocytosis of the neurotoxin. The heavy and the light chain of botox are cleaved. The light chain translocates to the cytosol and cleaves the C-terminal of the SNAP-25 protein. This inhibits SNARE complex formation and therefore inhibits neurotransmitter release (Blumenfeld et al. 2010b), such as substance P, calcitonin gene-related peptide (Blumenfeld et al. 2010a) and glutamate from the peripheral termini of primary afferents. Botox® inhibits peripheral signals to the central nervous system and thus indirectly inhibits central sensitization (Robertson & Garza 2012).

Our study shows several limitations, such as the retrospective study design and the rather small sample size. As well, the quantity of available MRIs might be too small, not everyone of our migraine patients between 2002 and 2009 underwent a MRI. Our definition of responders and non-responders, despite being very pragmatically and close to the clinical context, may contribute to some false results: first, patients in the non-responder group (one or two treatments) could be “cured” of migraine for a certain time. Second, patients with a very long treatment interval were included in the study, ending in 2009. Third, patients, corresponding to a treatment, but unable to pay for further treatments. Some false results in the responder group (≥ three treatments) could arise, first, from non-responders, having tried several times Botox®. However, more than three treatments without any sort of response are very unlikely. Second, an initial responder becomes a non-responder.

Improvements could be obtained by carrying on the study in a prospective design and by realizing more MRIs in our clinic.

## Conclusions

WML on MRI scans cannot serve as a marker to predict a positive response to Botox®. The meaning of the WML in the migraine population remains unclear, being probably not of clinical importance. But they are often a sign for migraine chronification and longer lifetime history of migraine. They can be seen as well in other clinical conditions like cerebrovascular diseases, different types of dementia, inflammatory diseases and bipolar depression, which can be important comorbidities to migraine. They have to be considered while having a look at white matter lesions in the context of migraine.
